# Characterizing PFASs in aquatic ecosystems with 3D hydrodynamic and water quality models

**DOI:** 10.1016/j.ese.2024.100473

**Published:** 2024-08-08

**Authors:** Jingjie Zhang, Huiting Chen, Nguyen Viet Tung, Amrita Pal, Xuan Wang, Hanyu Ju, Yiliang He, Karina Yew-Hoong Gin

**Affiliations:** a State Key Laboratory of Black Soils Conservation and Utilization, Northeast Institute of Geography and Agroecology, Chinese Academy of Sciences, Changchun, 130102, China; b Key Laboratory of Wetland Ecology and Environment, Northeast Institute of Geography and Agroecology, Chinese Academy of Sciences, Changchun, 130102, China; c Department of Civil & Environmental Engineering, National University of Singapore, 117576, Singapore; d National University of Singapore, Environmental Research Institute, 5A Engineering Drive 1, 117411, Singapore; e Shenzhen Municipal Engineering Lab of Environmental IoT Technologies, Southern University of Science and Technology, Shenzhen, 518055, China; f School of Environmental Science and Engineering, Shanghai Jiao Tong University, Shanghai, 200240, China

**Keywords:** Emerging contaminants (ECs), PFASs, PFOA, PFOS, Fate and transport, Integrated modeling, Risk assessment

## Abstract

Understanding how per- and polyfluoroalkyl substances (PFASs) enter aquatic ecosystems is challenging due to the complex interplay of physical, chemical, and biological processes, as well as the influence of hydraulic and hydrological factors and pollution sources at the catchment scale. The spatiotemporal dynamics of PFASs across various media remain largely unknown. Here we show the fate and transport mechanisms of PFASs by integrating monitoring data from an estuarine reservoir in Singapore into a detailed 3D model. This model incorporates hydrological, hydrodynamic, and water quality processes to quantify the distributions of total PFASs, including the major components perfluorooctanoate (PFOA) and perfluorooctane sulfonate (PFOS), across water, particulate matter, and sediments within the reservoir. Our results, validated against four years of field measurements with most relative average deviations below 40%, demonstrate that this integrated approach effectively characterizes the occurrence, sources, sinks, and trends of PFASs. The majority of PFASs are found in the dissolved phase (>95%), followed by fractions sorbed to organic particles like detritus (1.0–3.5%) and phytoplankton (1–2%). We also assess the potential risks in both the water column and sediments of the reservoir. The risk quotients for PFOS and PFOA are <0.32 and < 0.00016, respectively, indicating an acceptable risk level for PFASs in this water body. The reservoir also exhibits substantial buffering capacity, even with a tenfold increase in external loading, particularly in managing the risks associated with PFOA compared to PFOS. This study not only enhances our understanding of the mechanisms influencing the fate and transport of surfactant contaminants but also establishes a framework for future research to explore how dominant environmental factors and processes can mitigate emerging contaminants in aquatic ecosystems.

## Highlights

• A 3D-PFAS model integrates hydrodynamic and fate processes on a catchment scale.
• PFASs are effectively modeled with a <40% deviation from measured concentrations.
• PFASs dominate in the dissolved phase (>95%), but gradually sink in sediments.
• The reservoir buffers PFOA risk under extreme (up to tenfold) external loadings.
• Aeration in the reservoir helps reduce the accumulation of contaminants.

## Graphical abstract

**Figure:**
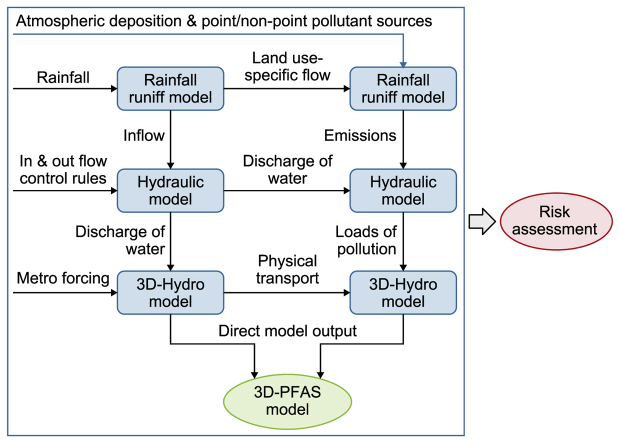

## 1 Introduction

Emerging contaminants (ECs) are compounds in different chemical classes, such as hormones, antibiotics, pesticides, surfactants, human and veterinary pharmaceuticals, and endocrine disruptors. Typically, they can be detected at concentrations ranging from 1 ng L^−1^ to 1 μg L^−1^ and are a potential threat to the environment and public health at higher concentrations [[1], [2], [3], [4]]. Recent studies have revealed the widespread occurrence of ECs in different water bodies and highlighted the problems of water resource management and the challenges of the environmental occurrences and treatment methods posed by ECs [[5], [6], [7], [8]].

Per- and poly-fluoroalkyl substances (PFASs), surfactant contaminants commonly found in water, are compounds usually grouped into long- or short-chain-length carbon–fluorine-based compounds. PFASs are prominent ECs and have been industrially manufactured and extensively used for over 60 years in different commercial products, such as stain repellents, polishes, paints, and coatings. Among PFASs, perfluorooctanoate (PFOA) and perfluorooctane sulfonate (PFOS) are widely used, predominantly found in different environmental matrices and included in regulations [9,10]. Although 3M Company, North America's largest producer, has phased out PFOS-based products, PFOS is still being produced in many other countries [11,12]. Moreover, owing to their environmental persistence, they continue to be ubiquitous in the environment, even after being phased out from manufacturing. PFASs have potential biological effects on aquatic organisms and increase the risk of adverse health effects on humans [[13], [14], [15], [16]].

To describe the fate and transport of ECs, numerical models are useful for assessing changes in ECs in the environment and for effectively restoring aquatic ecosystems [[17], [18], [19], [20], [21], [22], [23]]. However, modeling the fate and transport of organic contaminants at the catchment or regional scale requires combining processes in different environmental media to determine the transport and deposition efficiency of the target contaminants [[24], [25], [26], [27]]. Both the transport and deposition of organic contaminants in an ecosystem are contingent on the pollutant's properties, including its partitioning between different environmental media, its biological and chemical reactivity within receiving water bodies, and the hydraulic/hydrological factors influencing the pollutant, along with the pollutant sources at the catchment scale.

Possible physical, chemical, or biological processes [3] that may be associated with the fate of EC processes are as follows: (i) atmospheric deposition/volatilization at the catchment scale [28]; (ii) hydrological processes through runoff and leaching [29,30]; (iii) physical/chemical/biological processes involved in the received water diffusion body, such as advection, diffusion, and vertical convective mixing related to hydrodynamic conditions; and (iv) sorption/desorption, sedimentation and resuspension, hydrolysis, photolysis, degradation, transformation, bioaccumulation, and transformation processes in the water quality/ecological model. Other physical/chemical and biological processes such as the settling of inorganic particulate components and different phytoplankton species, reaeration of dissolved oxygen, decomposition of organic matter, nitrification/denitrification, and nutrient uptake in phytoplankton are considered in the water quality model. Theoretically, the modeling of the fate of ECs should consider all the abovementioned processes.

Considering these requirements, two main modeling approaches are commonly used: (i) multimedia box models [31,32] and (ii) spatially and temporally explicit multimedia mass balance models [33,34]. Multimedia box models consider a static environment that leads to low spatial and temporal resolution, while the latter models improve the static environment by including a dynamic simulation of flows and circulation models [35,36]. However, most models only address point sources without considering non-point pollutant loads and hydraulic and hydrological impacts at the catchment scale. Ignoring non-point pollutant loads can lead to a high underestimation of pollutant loadings, particularly in tropical rainforest regions such as Singapore, characterized by high annual rainfall and humidity [37]. Non-point pollutant loads from atmospheric dry and wet deposition as well as from urban runoff (e.g., pollutant sources from sewer leakage, residential areas, construction sites, roads, and park and recreation areas) have a significant impact on pollutant loadings [38].

In aquatic ecosystems, process-based models have been extensively used to better understand the fate and behavior of ECs [22]. Tong [3]conducted a comprehensive review of the advantages and limitations of the currently applied EC models. The Delft3D water quality modeling suite, as one of the modeling tools, has been extensively used for describing nutrient cycles, phytoplankton dynamics, and ecosystem function in freshwater and marine water systems in temperate regions [20,[39], [40], [41], [42], [43], [44], [45], [46], [47]]. Lindim [35] implemented STREAM-EU (Spatially and Temporally Resolved Exposure Assessment Model for European basins) in the Delft3D-WAQ modeling framework to simulate the environmental fate of PFOS and PFOA in the Danube River basin. The predicted PFOS and PFOA concentrations agreed well with concentrations measured using the STREAM-EU model [35]. Compared to other model tools, the Delft 3D modeling suite has been successfully applied in simulating general water quality parameters and studying the transport and fate of ECs in different water systems. The Delft 3D modeling suite is flexible, and most substances and parameters can be obtained from the existing library or added using the open process library. Moreover, it has a multispecies algal BLOOM model embedded in it, with more than 95% of parameter coefficients examined by many case studies. Furthermore, it can be easily integrated with other watershed models to address water quality issues at the catchment scale [48]. Moreover, very few case studies other than Delft3D water quality models have been applied to tropical areas, particularly concerning the modeling of ECs.

Herein, the Delft3D-WAQ application was extended using an integrated 3D-emerging contaminant model at the catchment scale to examine the possible fate and transport mechanisms of PFASs in a tropical water reservoir in Singapore. This integrated approach considered coupled physical/chemical/biological processes such as transport at the catchment scale, advection–diffusion, adsorption/desorption, settling of representative substances, growth, respiration/mortality of phytoplankton, and mineralization of organic compounds.

Compared to Lindim [35] approach, the dynamics of the distributions of PFOA and PFOS, as well as total PFASs in both the water column and the sediment based on differentiated partition coefficient log *K*_OC_ values, were considered in this study. Moreover, we included the functionality of hydrologic/hydrodynamic/water quality in the integrated modeling approach, which has direct/indirect impacts on the fate and transport of PFASs. Thus, the coupling of hydrological, hydrodynamic, eutrophication, and multiphase EC modules was proposed in our approach. The 3D-ECs (PFASs) model was built on the same hydrodynamic model as the water quality model and fed with the output of the water quality model. Moreover, the two particulate organic carbon (POC) components (i.e., algal and non-algal POC) from the 3D water quality model introduced in our study can be particularly useful for examining the impact of changes in nutrient and algal dynamics on the distributions of ECs in the future [21]. The integrated approach can model the impacts of rainfall–runoff, hydrological, and hydraulic dynamics on the transport of PFASs and other pollutants in the catchment; quantify emission loads and discharges to the reservoir; describe the direct/indirect impact of the hydrodynamics regime and change in the water quality on the dynamics of PFASs in the reservoir; and simulate the fate and transport mechanisms of ECs.

The objectives of this study are as follows: (i) introduce an integrated catchment-scale framework for modeling the fate and transport of ECs; (ii) apply the integrated model in describing process dynamics and quantifying the distribution of total PFASs and two major components (i.e., PFOA and PFOS) in water, sediments, and organisms in the reservoir; and (iii) assess potential risks of surfactant contaminant indicators for drinking water resources.

## 2 Study sites

### 2.1 Reservoir and its catchment

The reservoir, located in the heart of Singapore, aims to provide Singapore with a new source of drinking water and plays an important role in water-based recreational activities. The brackish water reservoir, fed predominantly by rainfall runoff through major inflow tributaries, was completely turned into a freshwater basin after constructing a barrage to separate seawater in 2009. The catchment includes five inflow tributaries. The southern part of the catchment represents an area of land south of the reservoir where only surface runoff directly feeds into the reservoir, with ∼2.4% of the total catchment area.

The primary sources of pollutants include wet and dry atmospheric deposition; runoff associated with bin centers and other urban facilities; runoff from parks and green zones, including fertilizers and animal wastes; erosion from construction sites, parks, and green zones; and possibly sewer leaks [38].

### 2.2 Sampling sites

**Fig. 1:**
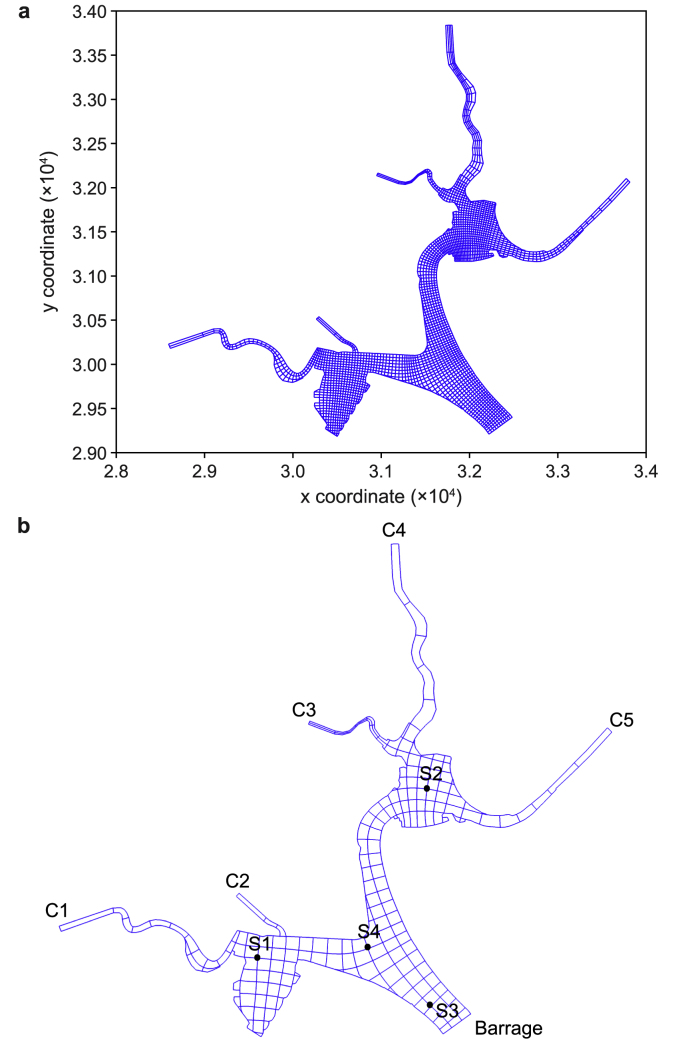
**a**, Grid map of hydrodynamic model. **b**, Aggregated grid map of the water quality model with its five tributaries (C1–C5) and four observation stations (S1–S4).

Sampling sites where PFAS concentrations in water, suspended solids (SS), and sediment cores were monitored from 2009 to date were similar to those used in previous studies [49,50]. Samples were collected at each tributary (C1, C2, C3, C4, and C5) and at four locations in the reservoir (S1, S2, S3, and S4). S1 receives input from C1; S2 from C3–C5; S3 is close to the barrage; and S4 is at the confluence of the five tributaries (C1–C5) (Fig. 1). The field dataset between 2009 and 2010 was used for model development, and the model results were confirmed using datasets collected between 2013 and 2014.

## 3 Modeling

### 3.1 Integrated water quality modeling

**Fig. 2:**
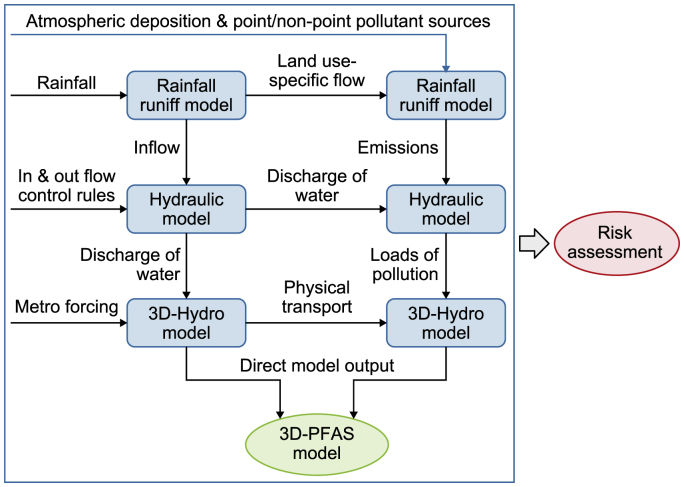
Integrated modeling approach at the catchment scale.

The modeling framework comprises the integration of six models (Fig. 2). The spatial schematics of the sources, the drains, and the reservoir are the essential inputs for the models. The level of detail and the accuracy of these schematizations largely determine how realistic simulations can be. The rainfall-runoff model (Sobek) and the generic emission model (EM) are based on the catchment's land use. These models generate the loads of water and substances that enter the drains and reservoirs. Note that EM considers both point sources and diffuse sources.

The 1-dmensional (1D) hydraulic model (Sobek-FLOW) provides water flow through the drains into the reservoir. The 3-dmensional (3D) hydrodynamic model (Delft3D-FLOW) simulates water flow horizontally and vertically, followed by dispersion within the reservoir. Moreover, it considers both thermal and salinity stratification. The hydraulic and hydrodynamic models can be coupled offline and online, where the latter provides data to the former based on which the flow through the gates in the dam is controlled.

Simulated water flows are delivered to the emission, 1D transport models (Sobek-WAQ/ECO), and 3D water quality models (Delft3D-ECO). Loads of substances obtained from the catchments are transported into the 1D model through the drains. The quantities of water and pollutants are estimated using the rainfall–runoff model and EM, respectively. In addition to PFASs, the 3D water quality model computes the concentration patterns and mass flows of nutrients, phytoplankton, organic matter, dissolved oxygen, sediment, salinity, and coliform bacteria in the reservoir's water and top sediment layers. The modeling framework produces year-long simulations to capture seasonal differences and allow for long-term predictions. The output of the integrated water quality model, such as total organic carbon (TOC), phytoplankton, and SS, are treated as forcing functions and inputs to the 3D-PFASs model. Moreover, the outputs of the EC model form the basis for assessing potential risks. Herein, our focus is primarily on the 3D-EC (3D-PFAS) model.

### 3.2 The 3D-PFAS model

This model was built on the previously developed hydrodynamic model with a 4 × 4 aggregated version of the original 2500 curvilinear grids (Fig. 1). The model has four layers in the water column and one layer in the sediment column. As per the integrated modeling approach, it is coupled with the hydrodynamic and water quality models and covers the PFAS loadings from five tributaries and atmospheric deposition (Fig. 2).

***State variables*.** The main state variables are described in Table 1.

**Table 1:** Main state variables in the water and sediment column.

State variables	Description
*Water column*	
Total_PFOA Total_PFOS Total_PFASs	Summation of the concentrations of PFOA/PFOS/all PFASs in the dissolved phase and particulate phase in the water column (ng L^−1^)
Dis_PFOA Dis_PFOS Dis_PFASs	Concentration of the dissolved PFOA/PFOS/all PFASs in the water column (ng L^−1^)
DOC_PFOA DOC_PFOS DOC_PFASs	Concentration of PFOA/PFOS/all PFASs attached to the dissolved organic carbon (ng L^−1^)
Fr PFOA/PFOS/PFASs Dis/PHYT/POC/DOC	Fractions of PFOA/PFOS/all PFASs distributed in the dissolved phase, phytoplankton, POC_noalgae (POC not including algae) and DOC (dissolved organic carbon)
*Sediment column*	
Total_PFOAS1; Total_PFOSS1; Total_PFASS1	Summation of the concentrations of PFOA/PFOS/all PFASs in the dissolved phase and particulate phase in the sediment column (ng m^−2^)
Dis_PFOAS1; Dis_PFOSS1; Dis_PFASS1	Concentration of the dissolved PFOA/PFOS/all PFASs in the sediment pore water (ng L^−1^)

***Forcing functions*.** The 3D-PFAS model used temperature, light, wind, and point/non-point loadings of SS, POC_noalgae, DOC, phytoplankton (algae), and IM1 and IM2 (inorganic matter 1 and 2) from the water quality model and flow rates from the hydrodynamic model as forcing functions.

***Process.*** The model depicted the primary processes of transport, mineralization of organic matter, adsorption, degradation, and uptake by algae through the following equations.

The emerging 3D-PFAS model is based on the DELWAQ 3D advection–diffusion–reaction equation. This equation is similar to that employed for modeling other pollutants, such as those in the eutrophication model (equation (1)) [51]; https://content.oss.deltares.nl/delft3dfm2d3d/D-Water_Quality_User_Manual.pdf)).

(1) $$\frac{\partial C}{\partial t}+u\frac{\partial C}{\partial x}+v\frac{\partial C}{\partial y}+w\frac{\partial C}{\partial z}=D\left(\frac{\partial^{2}C}{\partial x^{2}}+\frac{\partial^{2}C}{\partial y^{2}}+\frac{\partial^{2}C}{\partial z^{2}}\right)+S+R\left(c,t\right)$$

where *C* is the concentration of the simulated contaminants, and *D* is the diffusion coefficient. *S* is the waste load, including point and non-point loads and atmospheric depositions (see Section 2.1), estimated using emission and transport models (Fig. 2). *R*(*c, t*) is the reaction process, which may include physical processes (e.g., settling, resuspension, sorption, and desorption processes) and chemical and biological processes (e.g., degradation, mineralization, and conversions).

In natural environments, ECs transform a range of chemical or biochemical processes. Degradation rates in water systems can fluctuate considerably owing to diverse local conditions. Precisely quantifying specific degradation processes for a substance and comprehensively understanding all processes in the reaction term *R*(*c, t*) can be challenging. In this study, to simplify the simulation procedure, the overall degradation rates of ECs, including photolysis, hydrolysis, and biodegradation, are assumed to follow pseudo-first-order kinetics [21] A simplified mass balance (equation (2)) is employed, consistent with its current application in ECs [21,35,36,51]:

(2) $$M_{i}^{t+\Delta t}=M_{i}^{t}+\Delta t\times {\left(\frac{\Delta M}{\Delta t}\right)}_{\text{Tr}}+\Delta t\times {\left(\frac{\Delta M}{\Delta t}\right)}_{P}+\Delta t\times {\left(\frac{\Delta M}{\Delta t}\right)}_{S}$$

where Δt is the time step; $M_{i}^{t}$ and $M_{i}^{t+\Delta t}$ are the mass of the constituent for simulation at t and t+Δt; ${\left(\frac{\Delta M}{\Delta t}\right)}_{\text{Tr}}$ is the variation rate of the mass by transport from neighbor segments; and ${\left(\frac{\Delta M}{\Delta t}\right)}_{P}$ is the variation rate of the mass by physical processes (such as reaeration, settling, and adsorption processes), chemical processes (such as mineralization and degradation processes), or biological processes (such as uptake by algae), and ${\left(\frac{\Delta M}{\Delta t}\right)}_{S}$ is the variation rate of the mass by sources (waste loads).

Equation (2) has been extensively applied in modeling different water quality parameters (such as inorganic/organic components, nutrients, and phytoplankton species; https://content.oss.deltares.nl/delft3dfm2d3d/D-Water_Quality_User_Manual.pdf).

The transport process ${\left(\frac{\Delta M}{\Delta t}\right)}_{\text{Tr}}$ is dynamic and determined by the hypodynamic model, whereas the source term ${\left(\frac{\Delta M}{\Delta t}\right)}_{S}$ is dynamic and governed by boundary conditions or catchment loads. Herein, waste loads and boundary exchanges are estimated using the monitored water quality data from tributaries, thus establishing boundary conditions. Therefore, in this paper, only processes related to adsorption and degradation and specific to modeling ECs are discussed. For all other processes, please refer to the D-Water Quality Processes Technical Reference Manual (https://content.oss.deltares.nl/delft3d4/D-Water_Quality_Processes_Technical_Reference_Manual.pdf).

In addition to the impact of the hydrodynamic condition in the reservoir, the fate and transport of PFASs are primarily determined by the adsorption and degradation processes in both the water column and the sediment. In the solid phase, the adsorbed PFASs are controlled by the organic matter, which comprises dead POC, algae (ALG), and DOC in the water phase. In the 3D-PFAS model, different values of partitioning coefficients related to POC and algae (e.g., log KpocPFASs and log KalgPFASs for the water column; log KpocPFASsSl and log KalgPFASsS1 for the sediment) in different phases in both the water column and sediment are implemented (Table 2). The simulated total concentrations of PFASs and their two components, i.e., PFOA and PFOS, are described in equation (3):

(3) *C*_t_ = *C*_df_ *+ C*_doc_ *+ C*_poc_ *+ C*_alg_

where *C*_*t*_ is the total concentration of PFASs or one of their two components, i.e., PFOA and PFOS, including *C*_df_ (dissolved concentration), *C*_doc_ (adsorbed concentration to dissolved organic matter), *C*_poc_ (adsorbed concentration to dead particulate organic matter), and *C*_alg_ (adsorbed concentration to algae). The different fractions of the total concentration of PFASs and their two components are determined using equations (4), (5), (6), (7). Equations (8), (9) describe $K_{{\text{ppoc}}^{\prime }}$ and $K_{{\text{palg}}^{\prime }}$ as the two partition coefficients associated with the dead particulate organic matter and algae.

(4) $$f_{\text{df}}=\frac{\phi }{\phi +K_{{\text{ppoc}}^{\prime }}\times \left(C_{\text{poc}}+X_{\text{doc}}\times C_{\text{doc}}\right)+K_{{\text{palg}}^{\prime }}\times C_{\text{alg}}}$$

(5) $$f_{\text{doc}}=\left(1-f_{\text{df}}\right)\times \frac{K_{{\text{ppoc}}^{\prime }}\times X_{\text{doc}}\times C_{\text{doc}}}{K_{{\text{ppoc}}^{\prime }}\times \left(C_{\text{poc}}+X_{\text{doc}}\times C_{\text{doc}}\right)+K_{{\text{palg}}^{\prime }}\times C_{\text{alg}}}$$

(6) $$f_{\text{poc}}=\left(1-f_{\text{df}}\right)\times \frac{K_{{\text{ppoc}}^{\prime }}\times C_{\text{doc}}}{K_{{\text{ppoc}}^{\prime }}\times \left(C_{\text{poc}}+X_{\text{doc}}\times C_{\text{doc}}\right)+K_{{\text{palg}}^{\prime }}\times C_{\text{alg}}}$$

(7) $$f_{\text{alg}}=\left(1-f_{\text{df}}\;-f_{\text{doc}}-f_{\text{poc}}\right)$$

(8) $$K_{{\text{ppoc}}^{\prime }}=10^{\log\;K\text{ppoc}}\times 10^{-6}$$

(9) $${\;K}_{{\text{palg}}^{\prime }}=10^{\log\;K\text{palg}}\times 10^{-6}$$

In equations (4), (5), (6), (7), (8), (9), ϕ is porosity (V_water_/V_bulk_). $C_{\text{poc}}$, $C_{\text{doc}}$, and $C_{\text{alg}}$ are the concentrations of dead particulate organic matter, dissolved organic matter, and algae biomass, respectively, and described as the organic carbon concentration. $X_{\text{doc}}$ is the adsorption efficiency of DOC compared to POC; $K_{{\text{ppoc}}^{\prime }}$ and $K_{{\text{palg}}^{\prime }}$ are the partition coefficients used in the PFAS model for the dead particulate organic matter and algae, respectively.

**Table 2:** Primary parameters used in the 3D-PFASs model.

Parameters	Value	Description
VxSedPOCna (d^−1^)	2 × 10^−6^	Settling rate of particulate organic compounds
VxSedPhyt (d^−1^)	2 × 10^−6^	Settling rate of algae
VxDif0	1 × 10^−4^	Diffusion coefficient
*K*_0,deg_ (g m^−3^ d^−1^)	0	Zeroth order degradation rate
*k*_1,deg_ (d^−1^)	0.0001	Overall degradation of PFAS, PFOA, and PFOS in the water
Tc (°C)	2	Critical temperature for the degradation of PFAS, PFOA, and PFOS
*k*_t*,*deg_	1.07	Temperature coefficient loss of PFAS, PFOA, and PFOS in water
log *K*ppocPFASs	3.9	Log *K*_OC_ partition coefficient for total PFASs with the organic compound in the water
log *K*palgPFASs	3.9	Log *K*_OC_ partition coefficient for total PFASs with algae in the water
log *K*ppocPFASsS1	3.3	Log *K*_OC_ partition coefficient for total PFASs with organic compounds in the sediment
log *K*palgPFASsS1	3.3	Log *K*_OC_ partition coefficient for total PFAS with algae in the sediment
log *K*ppocPFOS	3.2	Log *K*_OC_ partition coefficient for PFOS with the organic compound in the water
log *K*palgPFOS	3.2	Log *K*_OC_ partition coefficient for PFOS with algae in the water
log *K*ppocPFOSS1	1.9	Log *K*_OC_ partition coefficient for PFOS with organic compounds in the sediment
log *K*palgPFOSS1	1.9	Log *K*_OC_ partition coefficient for PFOS with algae in the sediment
log *K*ppocPFOA	3.5	Log *K*_OC_ partition coefficient for PFOA with the organic compound in the water
log *K*palgPFOA	3.5	Log *K*_OC_ partition coefficient for PFOA with algae in the water
log *K*ppocPFOAS1	2.5	Log *K*_OC_ partition coefficient for PFOA with organic compounds in the sediment
log *K*palgPFOAS1	2.5	Log *K*_OC_ partition coefficient for PFOA with algae in the sediment
*X*_doc_	0.18	Adsorption efficiency of DOC relative to POC
SWPORH	0.66	Porosity in top sediment layers
ZResDM (gDM m^−2^ day^−1^)	194	Resuspension rate dry matter in the sediment S1 (gDM m^−2^ day^−1^)

In order to simplify the simulation procedure, all the processes, including photolysis, hydrolysis, and biodegradation, are merged into one overall degradation PFAS process. The degraded process of PFASs is the pseudo-first-order kinetics, described in equation (10), and a function of water temperature, presented in equation (11).

(10) $$R_{\deg}=R_{0,\deg},\text{if}\;T_{w}\;<\;T_{c}\text{,}$$

(11) $$R_{\deg}=K_{0,\deg}+\left(k_{1,\deg}^{20}\times k_{t,\deg}^{\left(T-20\right)}\times fr_{\deg}\times C_{t}\right)$$

where $R_{\deg}$ is the degraded rate (g m^−3^ d^−1^); Tc is the critical temperature for degradation; Tw is the water temperature derived from the hydrodynamic model; $k_{0,\deg}$ is the zeroth-order degradation rate (g m^−3^ d^−1^) (set as 0 in this study); $k_{1,\deg}^{20}$ is the first-order degradation rate at 20 °C (d^−1^); $k_{t,\deg}$ is the temperature coefficient of degradation; and $fr_{\deg}$ is the fraction subjected to degradation.

### 3.3 Model calibration and simulation

The integrated water quality model has been developed and calibrated previously against observations from April 1st, 2009, to December 31st, 2010 [data provided by the Public Utilities Board (PUB)]. Note that additional recalibrations and validations of the hydrodynamic and water quality model have been performed by the PUB [19]. The PFAS model was developed based on measured data between 2009 and 2010 (with very few available data). The estimated partition coefficients of log *K*_OC_ for total PFASs, PFOA, and PFOS used in the model were based on a lab study [52]. Note that a slight modification was made for the model calibration against the data between 2009 and 2010. Table 2 lists the values of the calibrated partition coefficients and other primary parameters used in the model. Calibration was performed using a standard trial-and-error procedure. The estimated partition coefficients based on the laboratory experiments were tested within a range of ±20% change. The default values of the remaining parameters used in the PFAFSs and its two components (PFOA and PFOS), which were not sensitive during the calibration, were selected from the existing library within the Delft3D software suite.

Four stations in the reservoir were selected for calibrating and validating the 3D-PFASS model. Data between 2013 and 2014 were used for model verification and validation. The hydrodynamic and water quality models were calibrated against data in 2010 and then validated based on data in 2013 and 2014. Herein, only the 3D-PFAS model is discussed and presented; for other models, please refer to our previous studies and PUB reports [19]. In the following subsections, the results are focused only on total PFAS and its two components, i.e., PFOA and PFOS. The contributions of PFASs from the five tributaries, treated as boundary loadings in the PFAS model, were estimated based on the measurements between 2013 and 2014. The PFAS model was run for five years, from January 1st, 2010, to December 31st, 2014.

The model performance was evaluated based on the average standard deviation (ARD) [53], which is expressed using equation (12):

(12) $$ARD=\frac{1}{n}\sum_{i=1}^{n}\left|\frac{X_{i,\text{sim}}-X_{i,\text{obs}}}{X_{i,\text{obs}}}\right|$$

where *X*_*i*,sim_ is the simulated value, *X*_*i,*obs_ is the observed value, and n is the number of observed points. The modeling performance is considered satisfactory if *ARD* is less than 25%.

## 4 Results and discussion

### 4.1 Calibration and validation

**Fig. 3:**
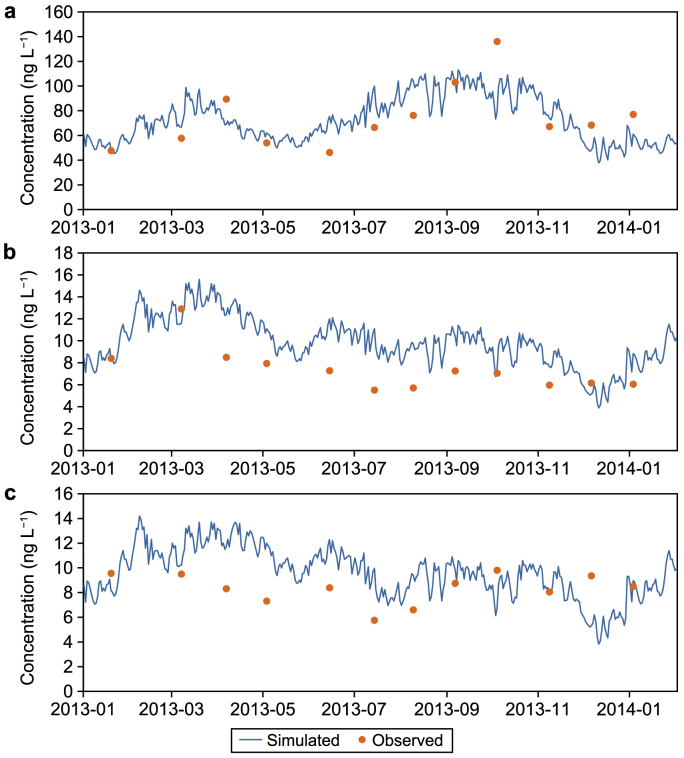
Comparison of observed (dots) and simulated (line) dissolved PFASs (**a**), PFOA (**b**), and PFOS (**c**) at S1 in the reservoir.

Simulated averages of total PFASs, PFOA, and PFOS in the water column between 2009 and 2010 were 45.4, 5.04, and 12.2 ng L^−1^, respectively (Supplementary Material Fig. S1). These values fall within the range of field measurements (total PFASs: 39.8–87.9 ng L^−1^, PFOA: 4.36–28.1 ng L^−1^, PFOS: 6.88–25.7 ng L^−1^). Fig. 3 shows the simulated and measured concentrations of dissolved PFASs and their two major components, PFOA and PFOS, in the water column at Station S1 from March 1st, 2013, to April 1st, 2014. The comparisons of the results for Stations S2 and S4 can be found in Figs. S2 and S3 (Supplementary Materials), respectively.

The results showed that the simulated dissolved PFAS concentrations in the water column reasonably matched with the average measured concentrations (as shown in Table 3 and Fig. 3, where the averaged deviations [53] were <40%) and captured the changing trends at Stations S1, S2, and S4. However, the predicted concentrations did not perfectly agree with the measured maximum and minimum values in the 2013–2014 simulation (Fig. 3), especially after January 2014, probably because of the 2013 hydrodynamic results repeatedly used for the water quality model simulation 2014. Another possibility is that the individual monthly measured value did not catch the peak or valley during that period. The mismatches of the peak and valley concentrations indicate that, at the time of sampling, PFASs in the water column were not in equilibrium among the different carriers (water, TOC, particulate, and phytoplankton). PFASs’ concentrations in water could be considerably affected by suspended particles [52], with some being irreversibly absorbed in the sediment depending on the chain length. Given the little information on the loading from the catchment monitoring program and the extremely low detection concentrations of PFASs, as well as PFOA and PFOS (i.e., at multiple ng L^−1^, which may be more prone to larger analytical errors), the results are considered generally acceptable.

**Table 3:** Average deviation of simulated dissolved PFASs, as well as PFOA and PFOS, compared to the measured values.

	PFASs	PFOA	PFOS
S1	6.5%	34.9%	16.9%
S2	0.6%	27.2%	13.5%
S4	1.8%	16.1%	2.9%

### 4.2 Temporal dynamics of total PFASs at different stations and different layers

**Fig. 4:**
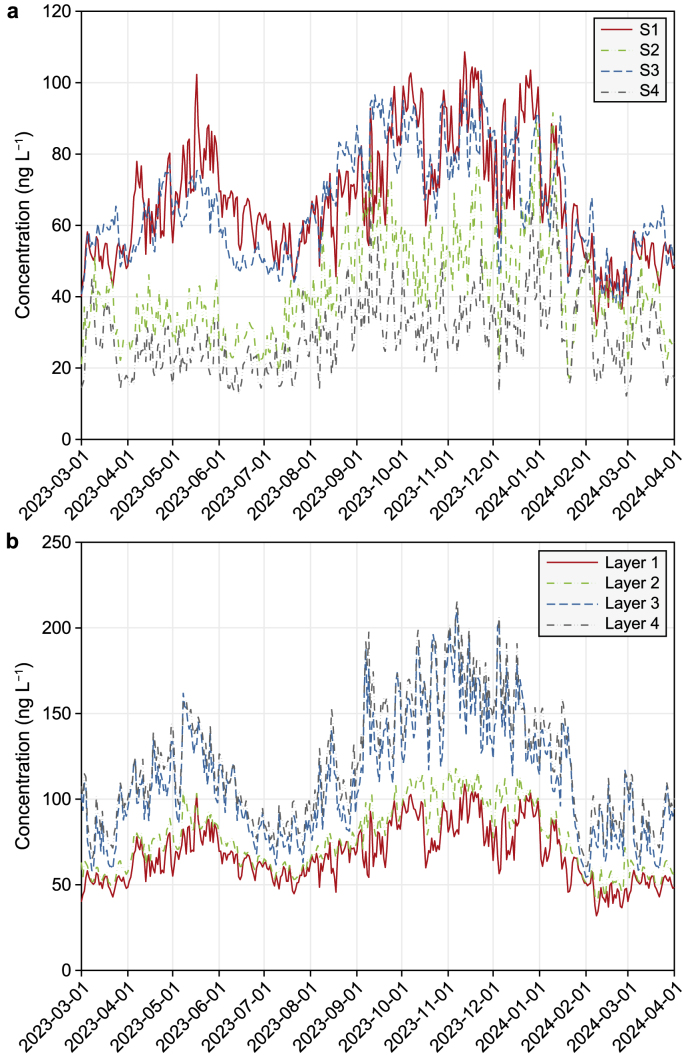
Time dynamics of total PFASs at the surface layer (Layer 1, **a**) at different stations and layers at a particular location (Station S2, **b**).

Fig. 4 shows the dynamic changes in the total PFAS concentrations (including dissolved PFASs, POC, DOC, and phytoplankton-associated PFASs) in the water column at different stations and layers. Only the results at one station's surface layer (Layer 1) are shown. Figs. S4 and S5 (Supplementary Materials) show the total dissolved PFAS concentrations at other stations and layers. The total dissolved PFAS concentrations ranged from 11.5 to 309 ng L^−1^ in the reservoir owing to the change in rainfall and runoff events over the one-year simulation period. High PFAS concentrations were reported in nearby tributaries (C1 and C4) from April to June and September to December 2013 (data not shown), accounting for the high reservoir concentrations, particularly close to tributaries at S1 and S2. Station S4 had a relatively low concentration at the confluence of the two adjoining river systems.

Fig. 4b shows the dynamics of total dissolved PFAS concentrations in different layers at Station S2 (other stations are shown in Supplementary Material Fig. S5). The results at Station S2 demonstrated that higher concentrations were observed in layers 3 and 4 (the bottom layers), primarily because the PFASs were carried by settling particles and exchanged with the accumulated concentrations in the sediment through resuspension and diffusion processes. A slight difference was observed at S3 near the barrage, where relatively higher mixing occurred owing to the operating aerators.

The two major components of PFASs (i.e., PFOA and PFOS) contributed ∼8.7–10.8% and 10.9–13.5% of the dissolved PFASs, respectively. In addition to total dissolved PFASs, the dissolved concentrations of PFOA and PFOS in the different layers of the water column also showed significant differences (Supplementary Materials Figs. S6 and S8). Furthermore, their concentrations varied differently during the year. Compared to total PFAS concentrations, similar distinct peaks were observed for PFOA and PFOS in 2013. PFOA demonstrated relatively higher concentrations from April to June 2013, the inter-monsoon period after the northeast monsoon season (Supplementary Material Fig. S6). The higher concentrations are attributable to the relatively weaker dilution during this period, suggesting that dilution is a major process influencing PFOA concentrations in the water column. However, the wet period between October and December 2013 may also yield high pollutant loading in the reservoir, resulting in relatively higher concentrations, as shown in Figs. S1, S3, and S5 (Supplementary Materials).

### 4.3 Fractions of PFASs in different phases of the water column

**Fig. 5:**
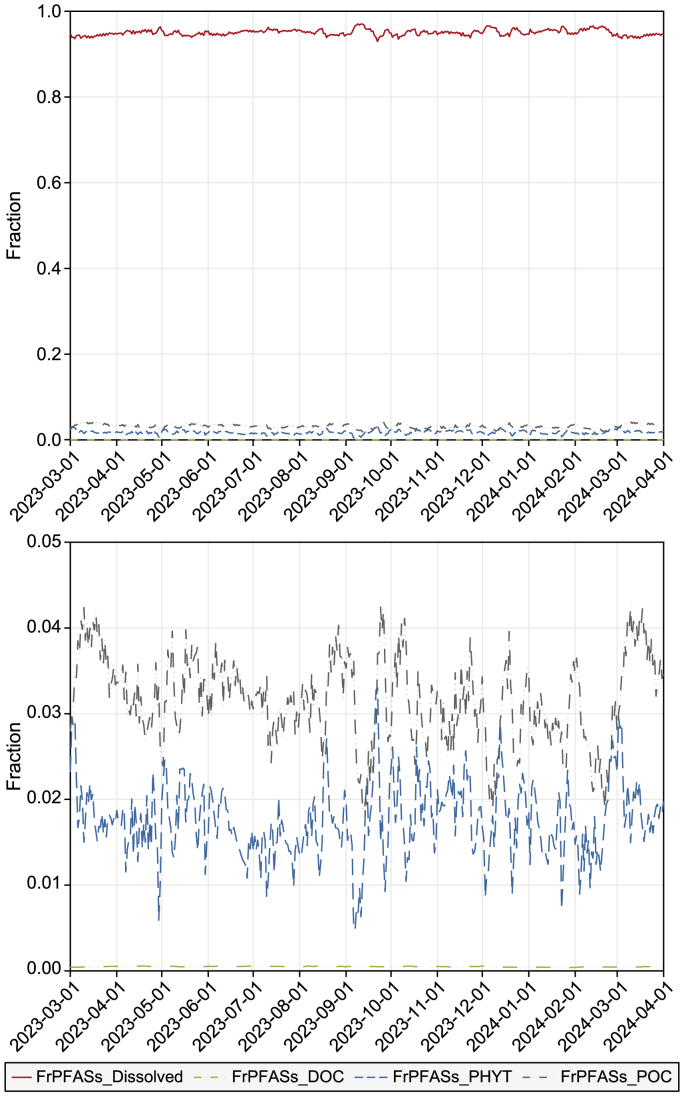
*Fractions of PFASs in different phases in the water column.* The upper figure shows the overall distribution, while the enlarged lower figure provides detailed trends for FrPFASs_DOC, FrPFASs_PHYT, and FrPFASs_POC within the fraction range of 0.00–0.05. *FrPFASs_Dissolved: the concentration fraction of PFASs in the dissolved phase; FrPFASs_DOC: the concentration fraction of PFASs associated with dissolved organic carbon in water; FrPFASs_PHYT: the concentration fraction of PFASs associated with phytoplankton in water; FrPFASs_POC: the concentration fraction of PFASs associated with particular organic compounds (no-algal part).*

Fig. 5 shows the fractions of PFASs in different phases in the water column. In terms of the total concentration in each phase in the water column, most PFASs were in the dissolved phase (>95%), followed by the fraction sorbed to organic particles such as detritus (1.0–3.5%) and the phytoplankton fraction (1–2%). Although some PFASs, especially those with relatively longer carbon chain lengths (6–10), could be sorbed to organic particles, those on the organic particles only accounted for a small amount of the total mass because of the considerably low mass of particles than water in the water column. The distributions of the proportions of PFASs in the different media were primarily determined by the perfluoroalkyl chain length [26].

A similar pattern of higher PFAS concentrations in the dissolved phase has been observed in other studies [54,55]. However, the importance of sorbed PFASs to organic particles cannot be ignored [56]. Higher concentrations of organic particles may lead to increased PFASs in the suspended solid phase. Previously, we reported that an increase in the algal biomass driven by the increased phosphorus concentration in the water column can change the fraction of distributed ECs between the dissolved and particle phases [21]. The fraction of free dissolved Bisphenol A (BPA) and N, N-diethyltoluamide (DEET) in the water column decreased, whereas that of ECs sorbed to the SS increased when the inorganic PO_4_^3−^ was increased to twice the external loading in the catchment. This increase induced the production of more phytoplankton, which acted as particles that increased the sorption capacity in the water column. Therefore, increasing the concentration of organic particles (especially phytoplankton) can reduce the risk of ECs by algae through the adsorption process. Herein, we report that when the inorganic PO_4_^3−^ loading in the catchment was doubled, the fraction of free dissolved BPA and DEET in the water column decreased, whereas that of ECs sorbed to the SS increased. This phenomenon was attributed to the increased production of phytoplankton, which acted as particles and enhanced the sorption capacity in the water column. This result indicates that increasing the concentration of organic particles can mitigate the risk of ECs by promoting adsorption processes facilitated by algae.

### 4.4 Changes in PFASs in benthic sediments

**Fig. 6:**
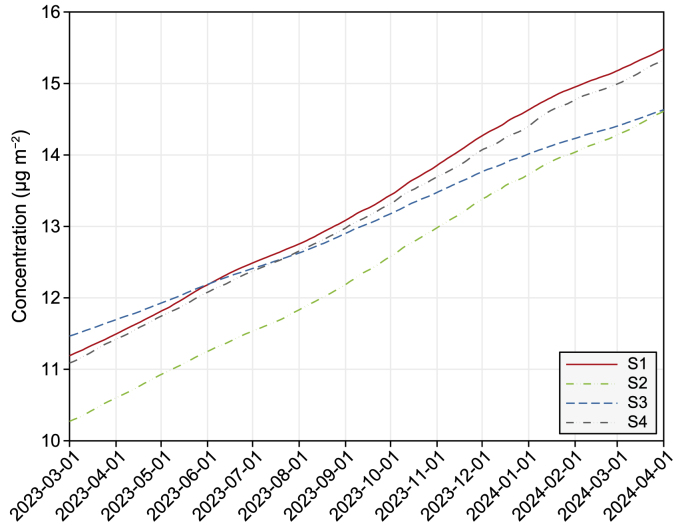
Simulated change in total PFASs in the sediment over one year.

The predicted changes in PFASs in the benthic layer show that total PFASs, PFOA, and PFOS in sediments increased over the years (Fig. 6; Supplementary Material Fig. S10). This result supports previous reports that sediments act as one of the two final sinks of PFASs [[57], [58], [59]]. Owing to the different loadings and hydrodynamic regimes, high concentrations were reported at Station S1 for PFASs, Station S2 for PFOA, and Station 4 for PFOS. The annual accumulations of PFASs, PFOA, and PFOS were approximately 4.3, 0.4, and 1.0 μg m^−2^, respectively. The smaller accumulated amount of PFOA in the sediment layer was attributed to its weaker sorption to the sediments [52,60]. The annual accumulation of PFASs in the sediments was lesser than that reported in the Lake Chaohu study, in which the annual sediment deposition was determined to be between 0.020 and 0.306 ng cm^−2^ [61].

### 4.5 Spatial distribution of total PFASs in the reservoir

**Fig. 7:**
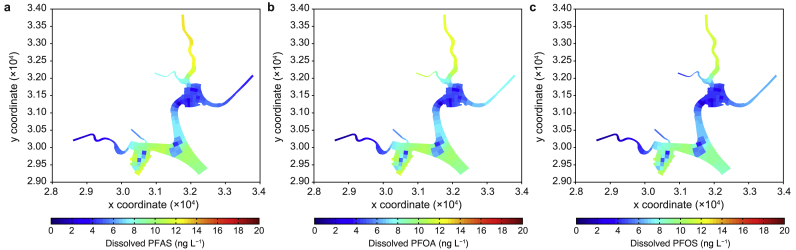
Spatial distributions of PFASs (**a**), PFOA (**b**), and PFOS (**c**) in the reservoir.

Fig. 7 shows the spatial distributions of total PFASs, PFOA, and PFOS concentrations in the reservoir at a single time point in November 2013 (random choice). The concentrations of PFASs and PFOA (Fig. 7a and b) were higher in the southwest and northern basins of the reservoir, reaching as high as 160 ng L^−1^. This result agrees with previous observations because of the higher loadings from the two river tributaries (C1 and C4).

The PFOS concentrations (Fig. 7c) in the reservoir simultaneously demonstrated the same spatial distribution pattern. Relatively higher concentrations of PFOS were reported in the southwest and northern parts of the reservoir because of the high loads from nearby catchments (C1 and C4).

### 4.6 Implications

To determine the potential risk of PFASs to aquatic organisms, the predicted no-effect concentrations (PNECs) have been examined based on the available acute toxicity data. Rostkowski, Yamashita [62] reported the lowest PNEC value of 50 ng L^−1^ for PFOS in water because of the bioaccumulation for trophic IV fish-eating birds. For PFOA, Hoke, Bouchelle [63] suggested a PNEC of 100 μg L^−1^ using an assessment factor of 1000 when extrapolating acute to chronic toxicity. Our simulation results demonstrated that PFOS and PFOA concentrations in the one-year simulation were less than 16 ng L^−1^. The risk quotients of PFOS and PFOA, which are calculated from the ratio of measured environmental concentration (MEC) (in this case, the simulated concentration) to PNEC, were <0.32 and < 0.00016, respectively, indicating the acceptable risk of PFAS in this water body.

**Fig. 8:**
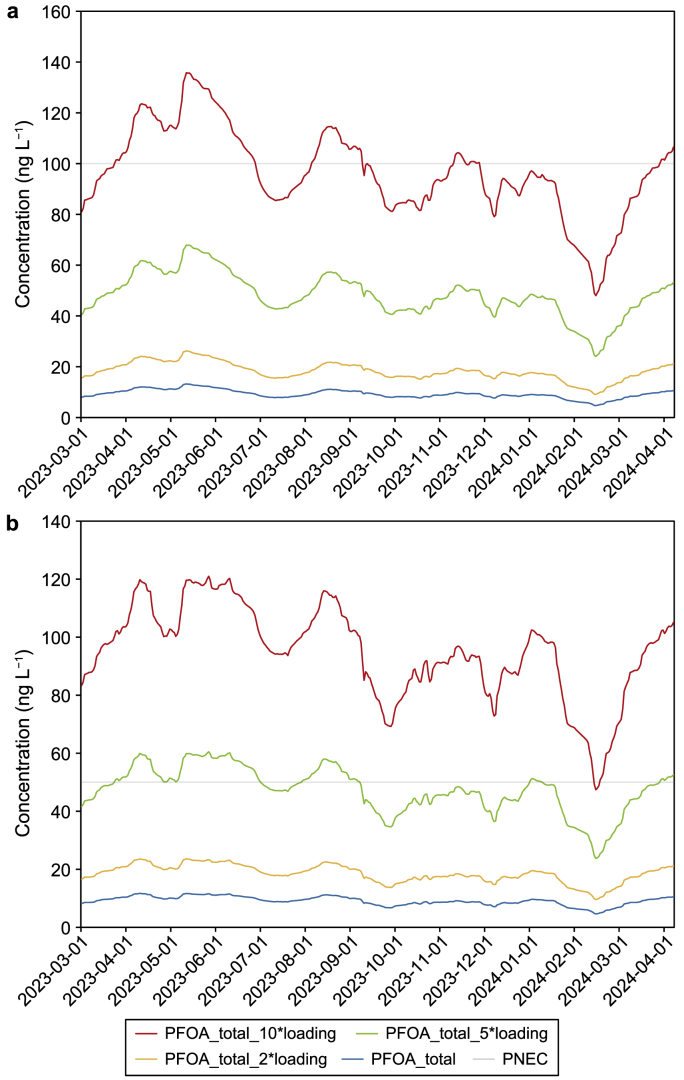
Comparison of predicted total PFOA (**a**) and PFOS (**b**) concentrations against their PNECs between the current loading and scenarios with two-, five-, and ten-times increased loadings in the reservoir.

To determine the potential risk of higher loadings in hypothetical scenarios, we tested three hypothetical scenarios involving a two-, five-, and ten-time increase in the current loading within the catchment. Subsequently, we predicted the corresponding concentrations in the reservoir. Fig. 8 compares the changed concentrations of PFOA and PFOS between the current loading and two-, five-, and ten-time loadings in the reservoir.

As the external loading was increased to twice its original amount, the predicted concentrations of the two major components, PFOA and PFOS, remained considerably lower than their PNEC values. Furthermore, a five-fold increase in the loading only sporadically resulted in PFOS exceeding its PNEC value, indicating the reservoir's robust buffering capacity in neutralizing the risk from these surfactant contaminants. Note that with a ten-fold increase in the loading, the predicted concentrations of PFOA only sporadically exceeded its PNEC value, whereas PFOS concentrations were nearly double their PNEC values. This underscores the reservoir's considerable buffering capacity, particularly for managing the risk associated with PFOA compared to PFOS.

**Fig. 9:**
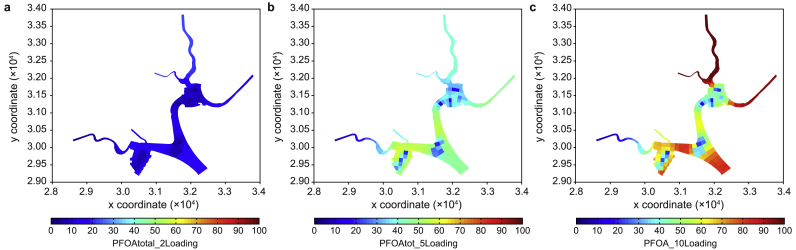
Predicted averaged PFOA concentrations with two- (**a**), five- (**b**), and ten-times (**c**) increased loadings in the reservoir.

**Fig. 10:**
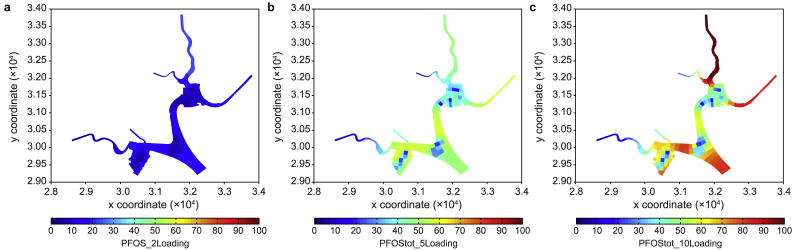
Predicted averaged PFOS concentrations with two- (**a**), five- (**b**), and ten-times (**c**) increased loadings in the reservoir.

The substantial buffering capacity in neutralizing the risk from PFOA and PFOS contaminants in the reservoir is reflected in the predicted averaged spatial distributions of the two PFAS components (Fig. 9, Fig. 10). In addition to a ten-fold increase in the current loading, their PNEC values were only exceeded in areas with high catchment loadings.

Relatively low concentrations were evident in the three areas where aeration was implemented (Fig. 9, Fig. 10b,c, light blue colored). This indicates that aeration in the reservoir could help reduce the accumulation of contaminants through potential mixing and dilution processes. Although acceptable risks were identified for external loading increases of less than five times based on the tested hypothetical scenarios, the accumulation of PFASs and their components in the sediment raises concerns about potential risks to benthic organisms.

### 4.7 Uncertainties and limitations

This study has certain uncertainties and limitations. First, model calibration depended on existing datasets, and additional sediment calibration is necessary, although the results agree with other reservoir studies. Moreover, recalibrating the hydrodynamic model with new datasets will be important in the future.

Second, we determined the water quality model's performance through continuity checks across various time steps and ultimately selected a 1-min time step for stability and accuracy. It is important to exercise caution when applying the coarser grid used in the water quality model aggregated from finer hydrodynamic model grids in other studies. Our recent study on the impact of implemented photovoltaic (PV) cells on surface water in reservoirs focused on using fine grids without aggregation in the hydrodynamic model to uphold the model's accuracy.

Third, in this study, the terms representing the loss of PFASs and their two components (PFOA and PFOS) because of chemical and biological processes (e.g., biodegradation, photolysis, hydrolysis, and transformation) were combined into a single degraded process. Future studies should focus on dissecting each process through a laboratory-based analysis to enhance model performance. Incorporating these results into the current model will refine its accuracy and reliability.

## 5 Conclusions

This study provides a comprehensive modeling of PFASs and their two major components (PFOA and PFOS) for an urban coastal water body. The integrated 3D water quality and PFAS model was developed and calibrated against data obtained between 2009 and 2010. It was then validated using field data obtained between 2013 and 2014, with acceptable results, where most of the relative average deviations were less than 40%. Subsequently, the model was applied to assess three scenarios regarding potential risks, indicating that the reservoir showed a high buffering capacity in mitigating the risk associated with PFOA compared to PFOS. The results demonstrated that the model could be useful to characterize the occurrence, sources, sinks, and trends of contaminant indicators (e.g., PFASs) and assess the potential risks in the water column and sediments in aquatic ecosystems. This approach helps understand the mechanisms that influence the fate and transport of ECs and provides a framework for future studies to explore how the dominant environmental factors change toward mitigating ECs in water bodies.

Our approach facilitates research to better understand the fate and transport of PFASs and their dynamic distributions in different media (solid and water phases) and explore interactions with other state variables, such as phytoplankton species and nutrients. Moreover, this approach can be applied to the sound management of PFASs and to examine other ECs by analyzing different scenarios and optimizing treatment measures in aquatic ecosystems. Continuous verification and improvement of the model performance are required with available datasets in the future.

## CRediT authorship contribution statement

**Jingjie Zhang:** Writing - Review & Editing, Writing - Original Draft, Supervision, Software, Methodology, Conceptualization. **Huiting Chen:** Writing - Original Draft, Visualization, Methodology, Data Curation. **Nguyen Viet Tung:** Writing - Review & Editing, Investigation, Data Curation. **Amrita Pal:** Writing - Review & Editing, Investigation, Data Curation. **Xuan Wang:** Writing - Review & Editing, Software, Formal Analysis. **Hanyu Ju:** Writing - Review & Editing, Visualization, Data Curation. **Yiliang He:** Writing - Review & Editing, Project Administration, Funding Acquisition. **Karina Yew-Hoong Gin:** Writing - Review & Editing, Project Administration, Methodology, Investigation, Funding Acquisition, Conceptualization.

## Declaration of competing interest

The authors declare that we have no financial interests or personal relationships that could have any influence on the work reported in this paper.
