# Supplementary material for: Characterizing PFASs in aquatic ecosystems with 3D hydrodynamic and water quality models
Source: Environ Sci Ecotechnol. 2024 Aug 8;22:100473. doi: 10.1016/j.ese.2024.100473 (PMC11381888; doi:10.1016/j.ese.2024.100473)
Supplement: Multimedia component 1 [file mmc1.docx]

**Characterizing PFASs in Aquatic Ecosystems with 3D Hydrodynamic and Water Quality Models**

Jingjie Zhang^a, b,c,d,e^, Chen Huiting^c^, Nguyen Viet Tung^c^, Amrita Pal^d^, Xuan Wang^c^, Hanyu Ju^a,b^, Yiliang He^f^, Karina Yew-Hoong Gin^c,d,^*

^a^ State Key Laboratory of Black Soils Conservation and Utilization, Northeast Institute of Geography and Agroecology, Chinese Academy of Sciences, Changchun 130102, China

^b^ Key Laboratory of Wetland Ecology and Environment, Northeast Institute of Geography and Agroecology, Chinese Academy of Sciences, Changchun 130102, China

^c^ Department of Civil & Environmental Engineering, National University of Singapore, Singapore 117576

^d^ National University of Singapore, Environmental Research Institute, 5A Engineering Drive 1, Singapore 117411

^e^ Shenzhen Municipal Engineering Lab of Environmental IoT Technologies, Southern University of Science and Technology, Shenzhen, 518055, China

^f^ School of Environmental Science and Engineering, Shanghai Jiao Tong University, Shanghai 200240, China

Supplementary Material

###
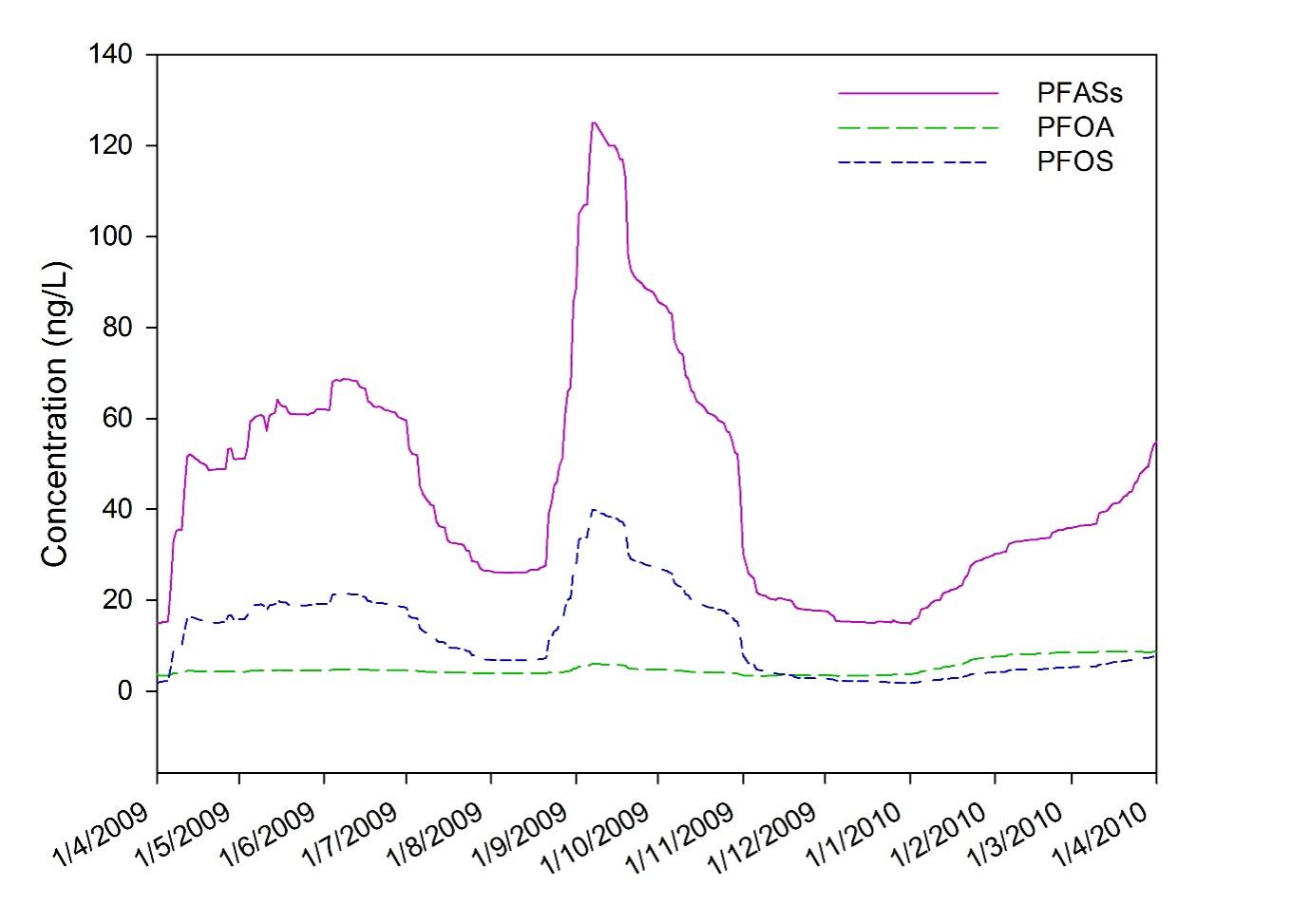


Figure S1 Simulated averages of total dissolved PFASs, PFOA and PFOS in the reservoir in 2009 and 2010.


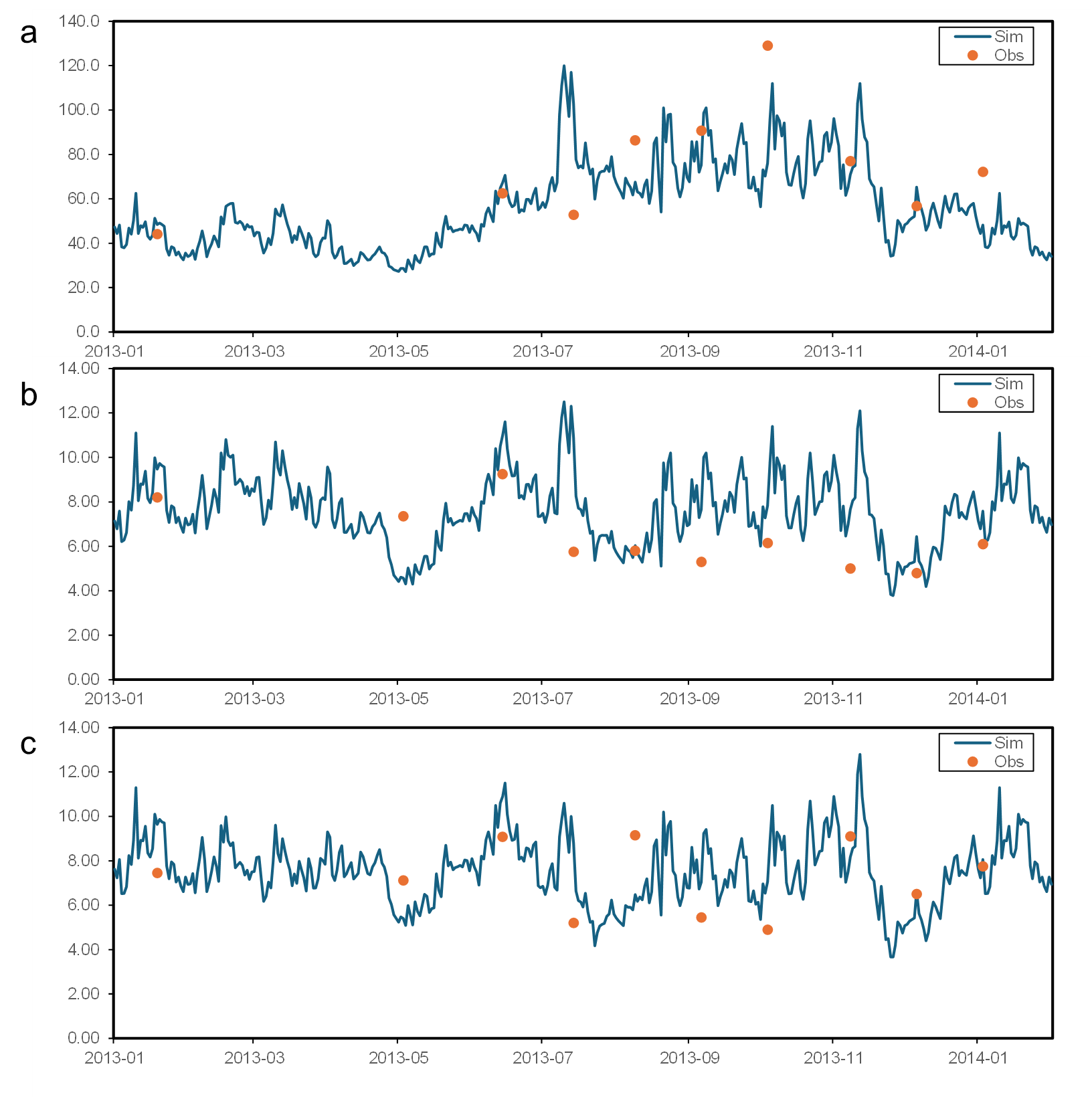


Figure S2 Comparison of measured (dots) and simulated (line) (a) dissolved PFASs, (b), PFOA, and (c) PFOS at S2 in the reservoir.


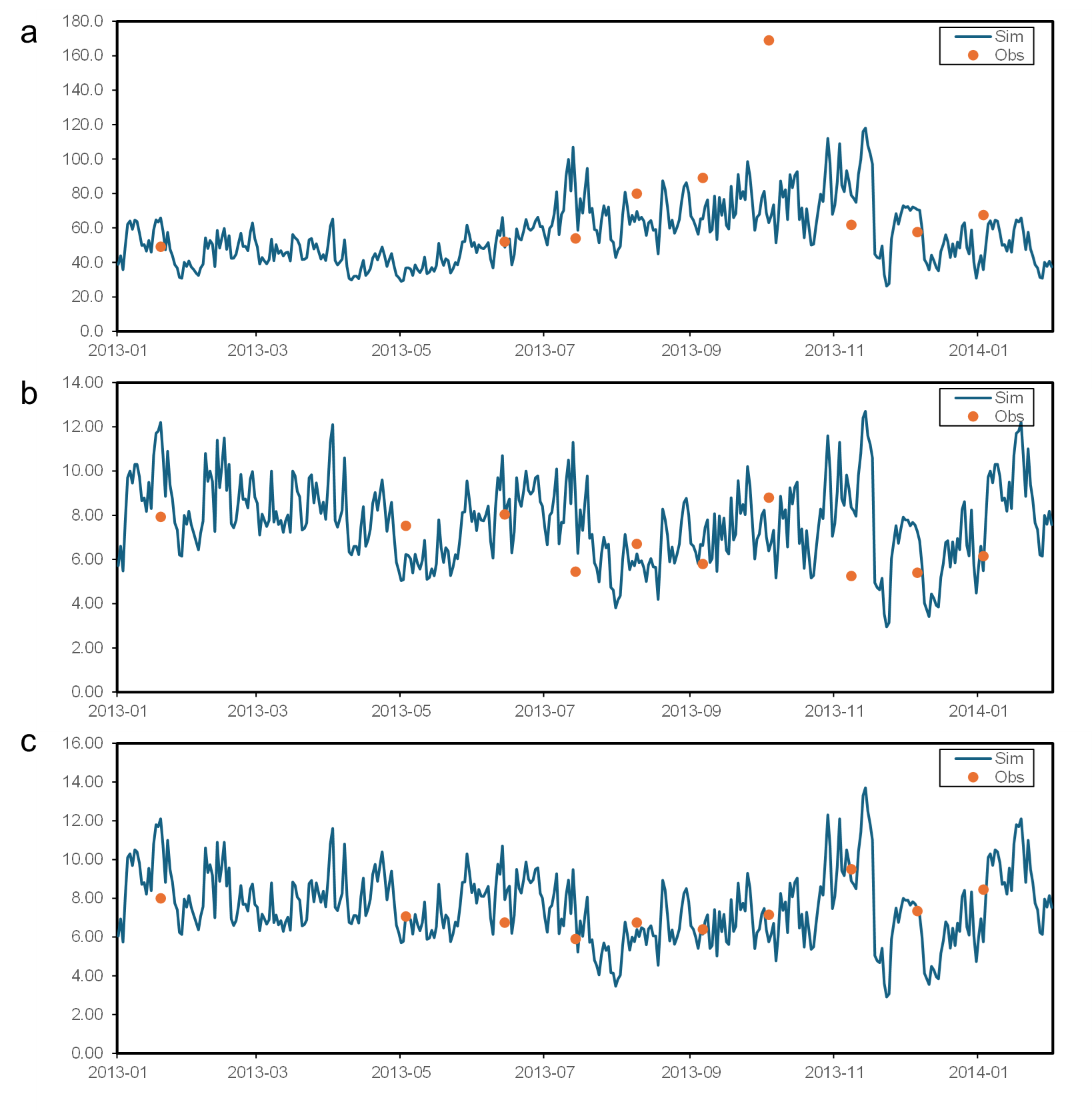


Figure S3 Comparison of measured (dots) and simulated (line) (a) dissolved PFASs, (b), PFOA, and (c) PFOS at S4 in the reservoir.


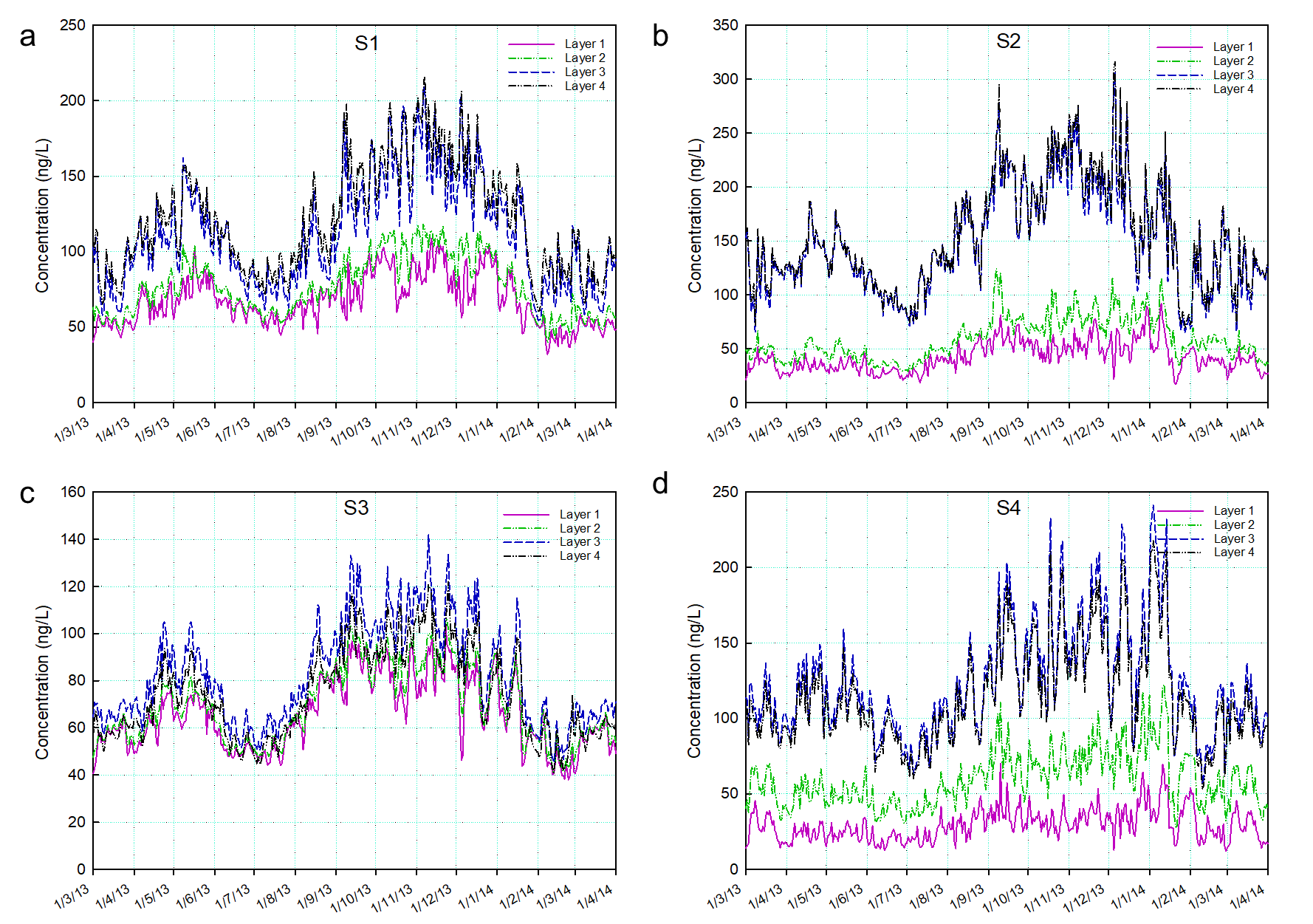


Figure S4 Time dynamics of total PFASs at different stations


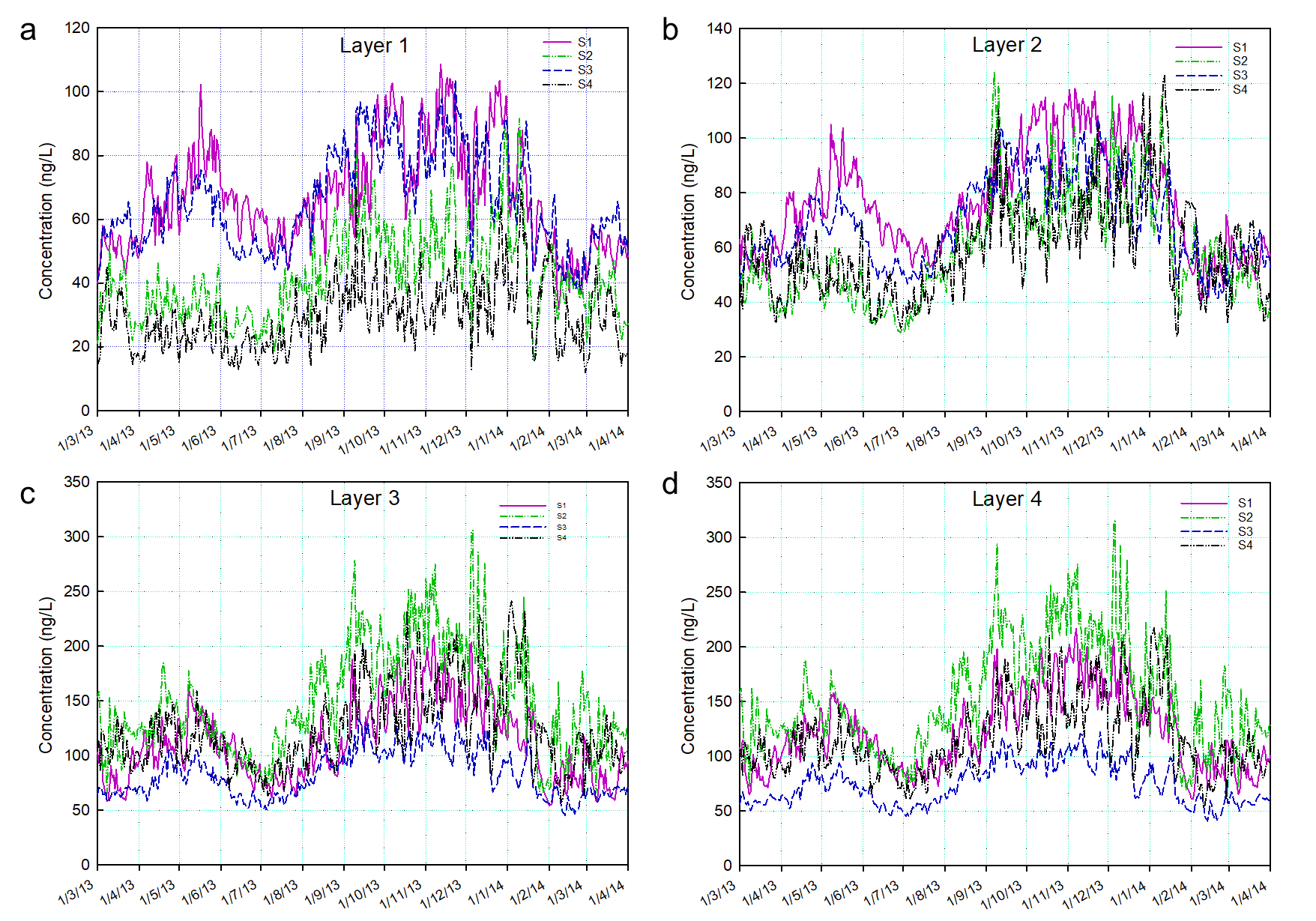


Figure S5 Time dynamics of total PFASs at different layers


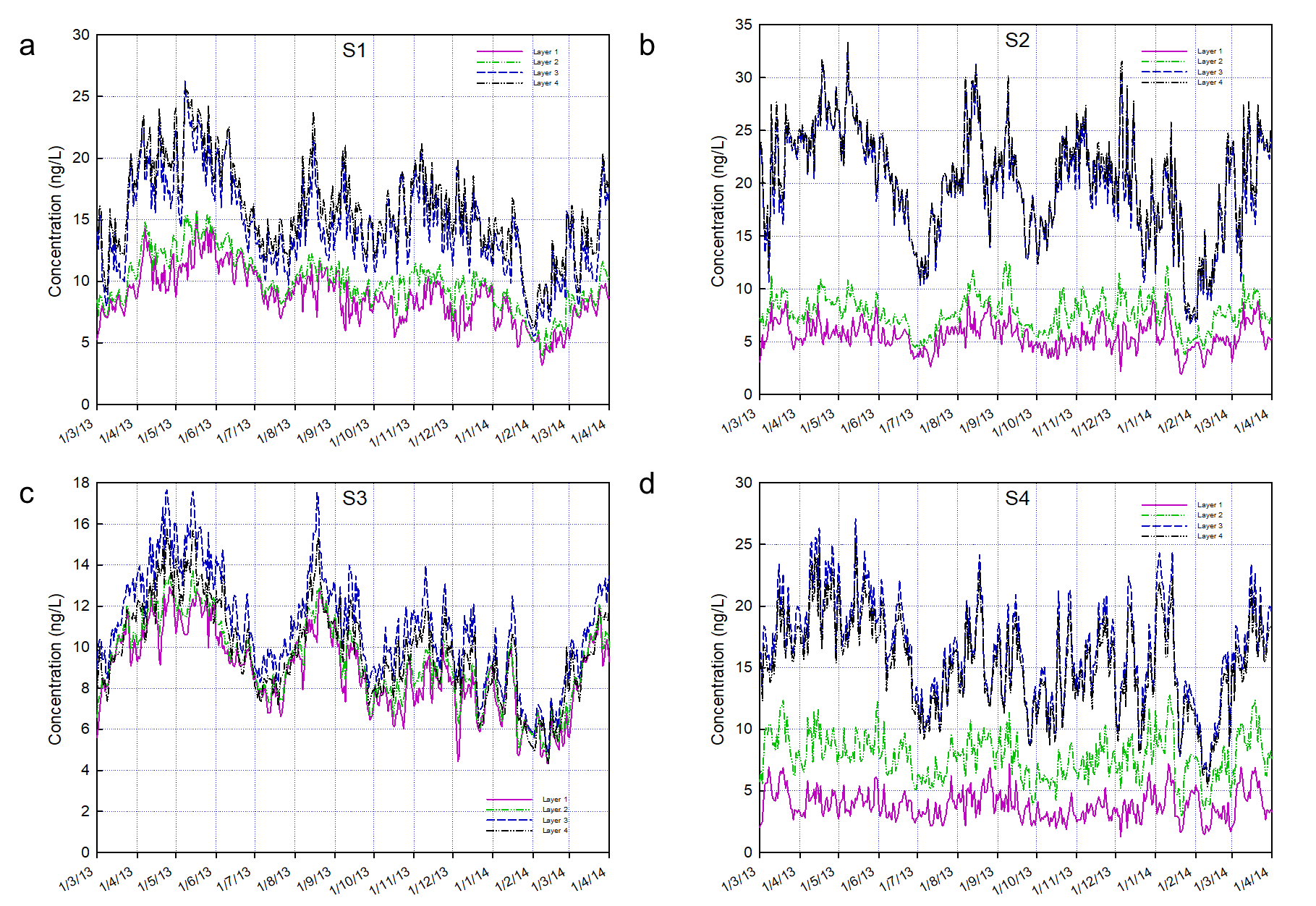


Figure S6 Time dynamics of total PFOA at different stations


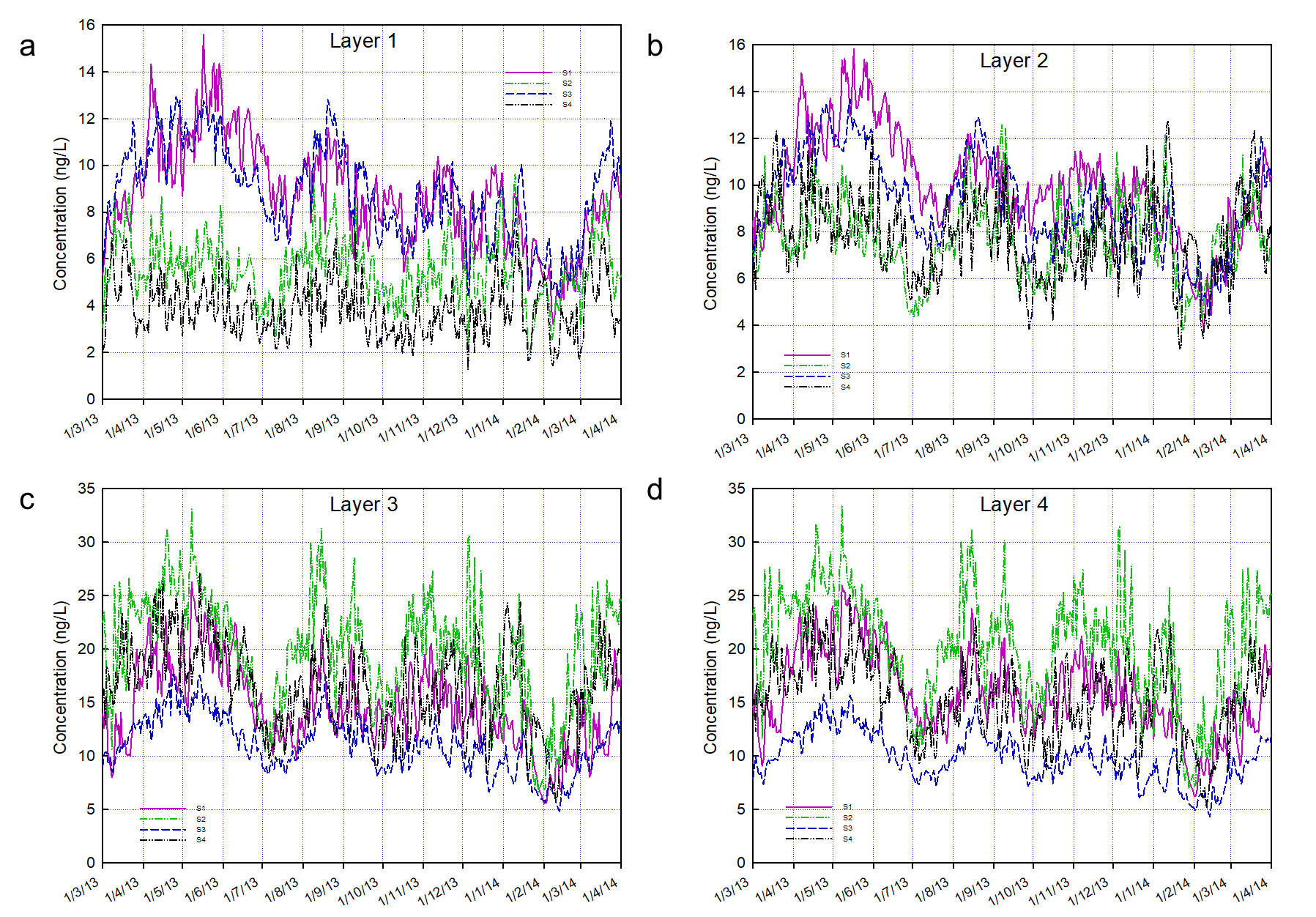


Figure S7 Time dynamics of total PFOA at different layers


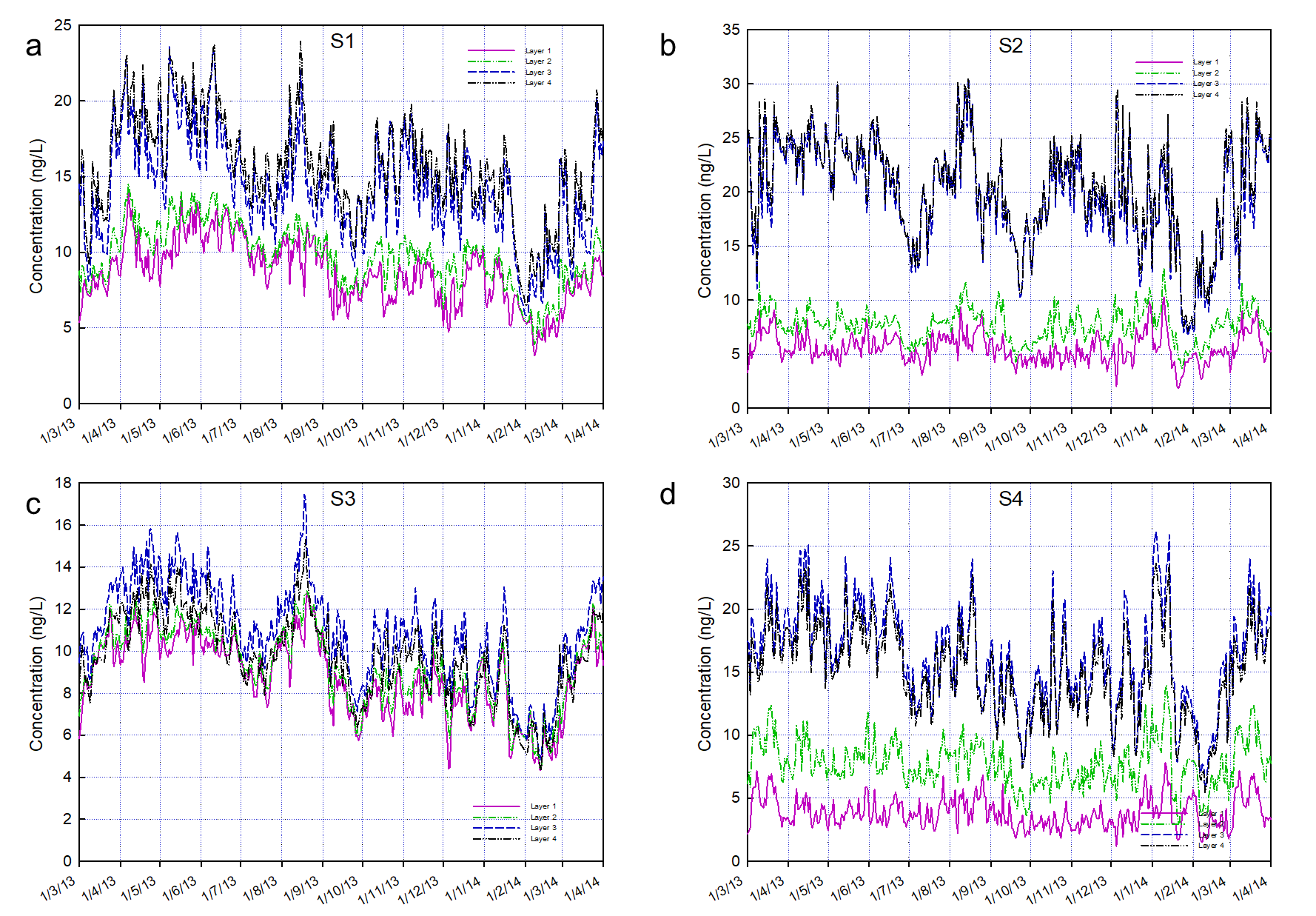


Figure S8 Time dynamics of total PFOS at different stations


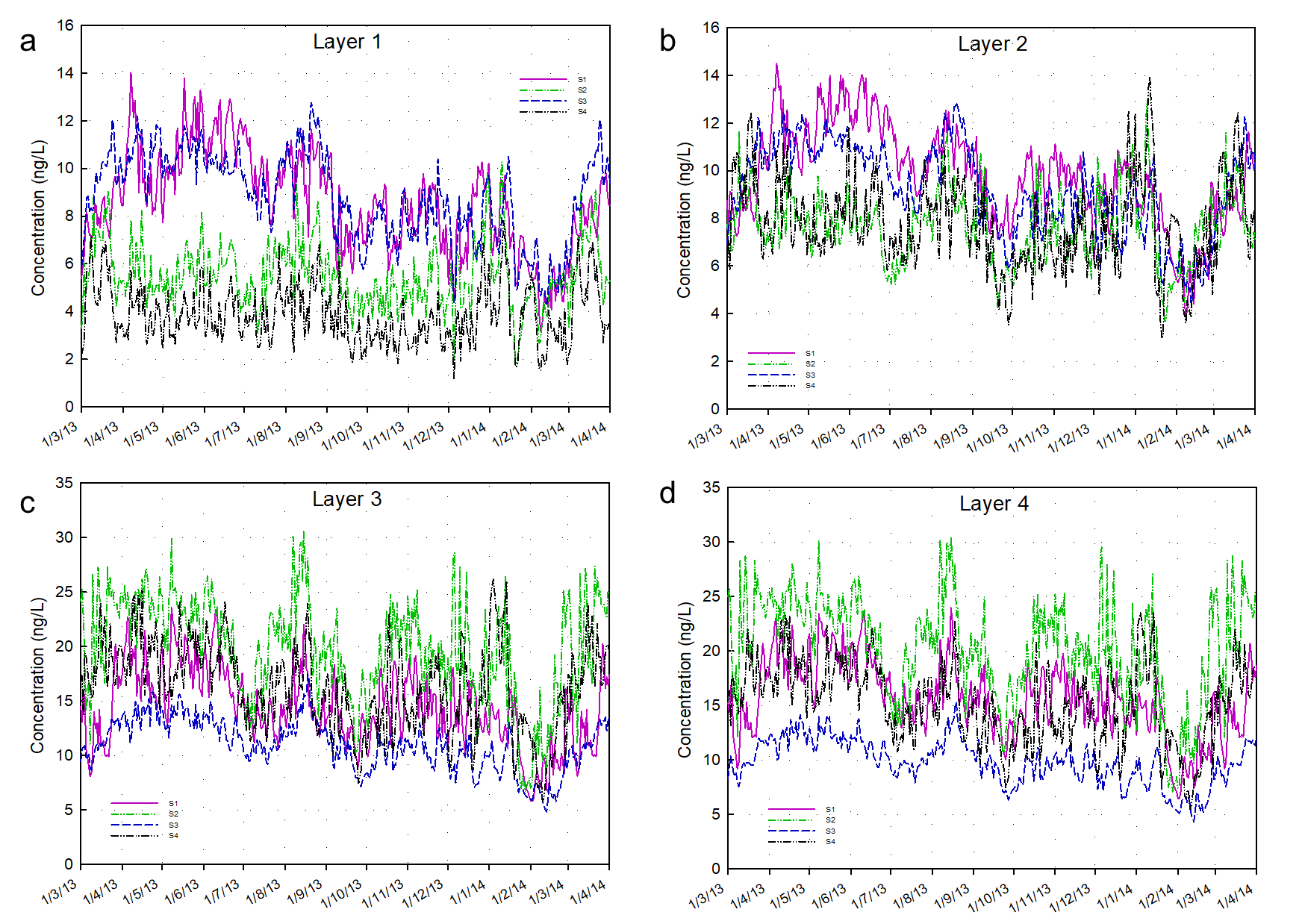


Figure S9 Time dynamics of total PFOS at different layers


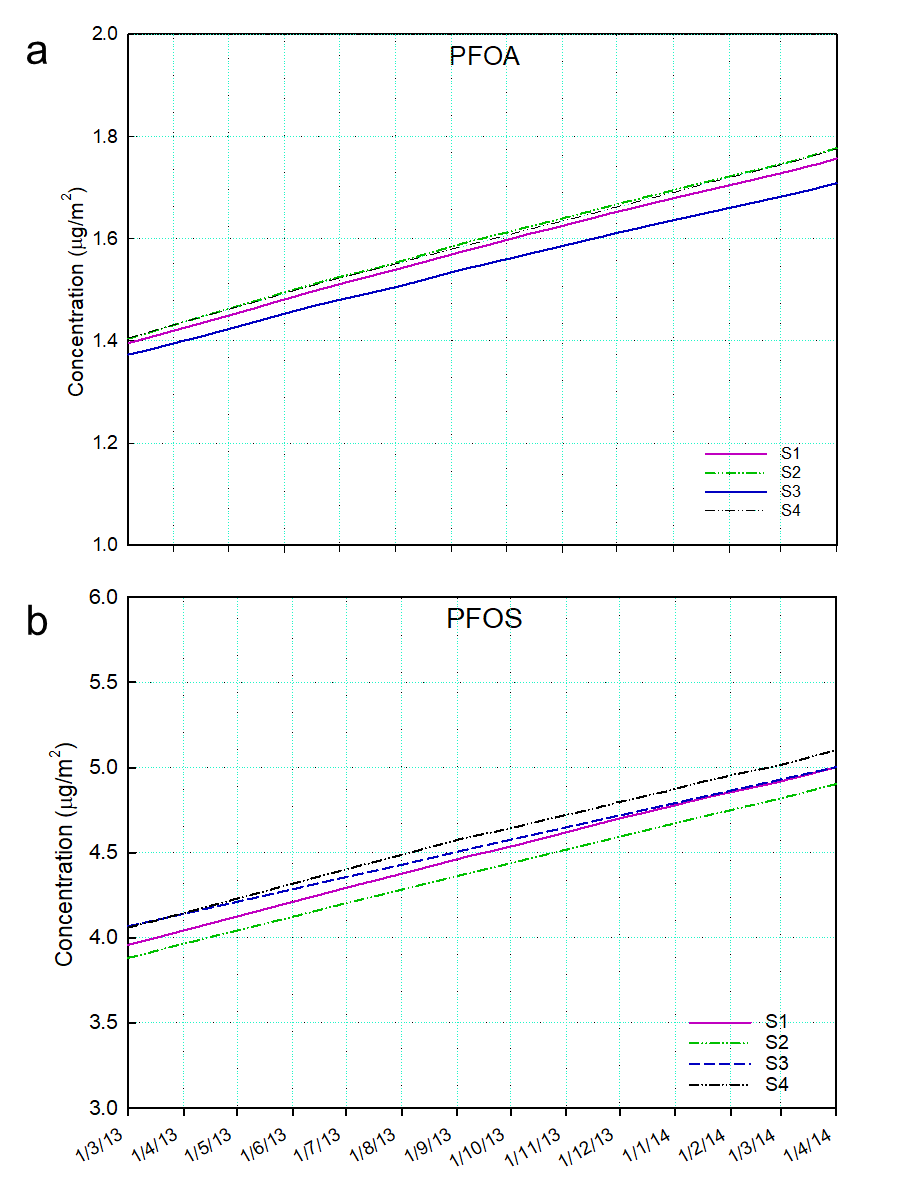


Figure S10 Simulated change in total PFOA and PFOS in the sediment over a one-year period
